# Analysis of Herbaceous Plant Succession and Dispersal Mechanisms in Deglaciated Terrain on Mt. Yulong, China

**DOI:** 10.1155/2014/154539

**Published:** 2014-10-23

**Authors:** Li Chang, Yuanqing He, Taibao Yang, Jiankuo Du, Hewen Niu, Tao Pu

**Affiliations:** ^1^MOE Key Laboratory of Western China's Environmental Systems, Collaborative Innovation Centre for Arid Environments and Climate Change, Lanzhou University, Lanzhou 73000, China; ^2^State Key Laboratory of Cryosphere Sciences, Cold and Arid Regions Environmental and Engineering Research Institute, Lanzhou 730000, China; ^3^University of Chinese Academy of Sciences, Beijing 100049, China; ^4^College of Earth and Environmental Sciences, Lanzhou University, Lanzhou 730000, China

## Abstract

Ecological succession itself could be a theoretical reference for ecosystem restoration and reconstruction. Glacier forelands are ideal places for investigating plant succession because there are representative ecological succession records at long temporal scales. Based on field observations and experimental data on the foreland of Baishui number 1 Glacier on Mt. Yulong, the succession and dispersal mechanisms of dominant plant species were examined by using numerical classification and ordination methods. Fifty samples were first classified into nine community types and then into three succession stages. The three succession stages occurred about 9–13, 13–102, and 110–400 years ago, respectively. The earliest succession stage contained the association of* Arenaria delavayi + Meconopsis horridula*. The middle stage contained the associations of* Arenaria delavayi + Kobresia fragilis*,* Carex capilliformis + Polygonum macrophyllum*,* Carex kansuensis*, and also* Pedicularis rupicola*. The last stage included the associations of* Kobresia fragilis + Carex capilliformis*,* Kobresia fragilis*,* Kobresia fragilis *+* Ligusticum rechingerana*, and* Kobresia fragilis + Ligusticum sikiangense*. The tendency of the succession was from bare land to sparse vegetation and then to alpine meadow. In addition, three modes of dispersal were observed, namely, anemochory, mammalichory, and myrmecochory. The dispersal modes of dominant species in plant succession process were evolved from anemochory to zoochory.

## 1. Introduction

Succession is regarded as the most important ecological concept apart from the ecosystem itself [1–3]. Repeated observations (e.g., through historical photography or long-term plot studies) have been employed to study plant temporal dynamics within a short period (e.g., from years to decades) [4–6]. However, for measuring temporal dynamics over longer time-scales (e.g., over several decades to centuries), space-for-time substitution has been used frequently [7]. Glacier foreland is considered as one of the most ideal places for examining ecological succession because it presents a temporal sequence of plants at a small spatial scale over a long period.

Matthews reviewed those pieces of research in relation to plant succession on glacier forelands over the past 100 years. He found that most of them are descriptive, while some of them are even anecdotal [7]. Quantitative analysis has been becoming popular in recent years, and more attention has also been paid to the investigation of plant succession processes [7], especially migration. Plant colonization and community assembly are formed via migration. So far, the most systematic investigation of seed dispersal was conducted on a glacier foreland in southern Norway [8, 9]. Yet, studies about the seed dispersal of dominant species within successional stages on glacier foreland and its associated mechanism are sparse. In addition, studies related to plant succession are mainly carried out in the European Alps [10–13], Scandinavia [14–17], and North America [4, 18, 19]. The study of plant succession on the glacier foreland in China is still in its infancy. At the moment, this kind of research has been only conducted on the foreland of Glacier No. 1 in the Urumqi River headwaters on Mt. Tianshan and the foreland of the Glacier Hailuogou on Mt. Gongga [20, 21].

Mt. Yulong is located in the southeastern margin of the Tibetan Plateau, which is also the southernmost snow-covered area in Eurasia. With its considerable diversity of habitats, Mt. Yulong supports abundant biological resources. It is considered as one of the three core clusters of endemic species in China. Because the south branch of the westerly wind controls the climate between November and April, the region is dry and prone to forest fire during the period. More than 600 km^2^ forest area between the altitudes of 3000–3800 m was affected by a large wildfire on Mt. Yulong in 1999, causing huge damage to the biological species there. Furthermore, subject to global climate warming, the frequency and severity of wildfire are predicted to further increase worldwide (e.g., the Mediterranean region [22]). Even the areas which are scarcely affected by wildfire are also influenced [23]. Under this situation, the risk of wildfire on Mt. Yulong is considered to be further increased in the future. Therefore, ecological restoration and reconstruction are urgently needed there. Baishui number 1 Glacier (hereafter Glacier-BS1) is the largest temperate glacier in this region and the temperate glacier is regarded as a most sensitive glacier to global warming [24]. Continuous observation and research for its change have been undertaken since 1999 [25–33]. Therefore, deglaciated terrain surrounding the glacier formed since the little ice age (between the 17th and 19th centuries) was selected as our study area and its slope distance is about 1250 m long from the glacier terminal (about 4350 m) to the altitude of 3800 m [24], with the unique environment at high elevation and low latitude. Subject to monsoonal climate, the terrain receives abundant precipitation, making it suitable for the growth of plants. Therefore, the study of plant succession in this area is important and sorely needed. Based on data collected from field observations in August 2011 and laboratory measurements, we analyzed the community composition and dispersal modes of dominant plant species on the foreland of Glacier-BS1 on Mt. Yulong. We sought to reveal the rate of plant succession and the variation of the dispersal modes of dominant plant species in this region. We hope the present study could serve as a theoretical reference for ecosystem restoration at a regional scale and a cross-check to other similar studies on the forelands in other glaciers [17]. In addition, the important determinants of plant succession were also discussed in this paper.

## 2. The Study Area

Mt. Yulong (26°59′–27°17′N, 100°04′–100°15′E) is located 25 km south of Lijiang City in northwestern Yunnan, China. The mountain stands in the southern margin of Hengduan Mountain of the Tibetan Plateau, which is the snow mountain closest to the Equator in Eurasia. The mountain has a width about 18 km from the west to the east and a length about 35 km from the south to the north and an elevation about 5596 m a.s.l. At present, there are fifteen glaciers on the mountain. The largest one is Glacier BS-1, which is 2.26 km in length, covering the area 1.32 km². The elevation of the glacier's equilibrium line is 4850 m.

In the little ice age, the lowest terminus of Glacier-BS1 was about 3800 m [24]. Temperature has been the principal factor affecting the development of Glacier-BS1, as rising temperature induces glacier ablation and mass loss [25–30]. The rate of annual mean temperature increase in the Hengduan Mountains is approximately equal to that of the globe (0.148°C/10 a) [32]. The glacier has retreated since the end of the 19th century, although the retreat is interrupted in some periods (Table 1). A long period of glacial advance occurred between 1957 and 1982 [24]. The terminus of the glacier had an elevation of 4365 m a.s.l. in 2011 [33].

We chose the foreland of Glacier-BS1 as our study area, which is the transition zone between alpine sub-arctic and alpine tundra climate. The alpine subarctic climate controls the zone between 3800–4000 m, with mean annual temperature of 3–7°C and precipitation of 1500–1800 mm. Although the average wind speed is not very high, the speed of gusts can often reach 18 m/s. The alpine tundra climate zone ranges from 4000–4300 m, with an average annual temperature of −4–2°C and covered by snow and ice. There are a variety of cold cushion plants, alpine meadow,* Rhododendron*, willow scrub, and subalpine dark coniferous forests (mainly spruce and fir) in this area.

## 3. Data and Methods

### 3.1. Field Sampling

**Figure 1:**
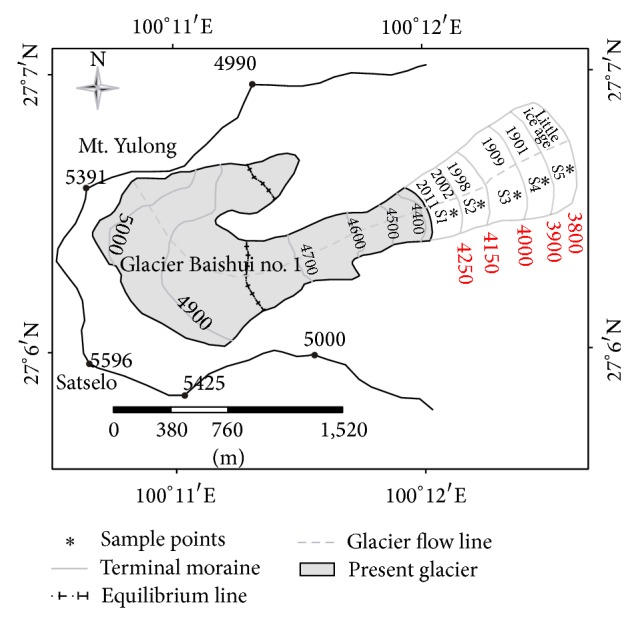
Map of sampling points at the Glacier-BS1 foreland on Mt. Yulong. ^*^S1–S5, represent five widely spaced sampling sites. Each site was sampled with ten quadrats. Solid blue lines represent the terminus of the glacier in 2002, 1998, 1909, 1901, and the 17–19th centuries.

By substituting space for time, a glacier foreland could be interpreted as a temporal sequence of ecological succession as the time for habitat development increases away from the margin of a retreating glacier [34]. Our samples were collected simultaneously at various parts of the deglaciated terrain of Glacier-BS1 of different ages. Five widely-spaced sites were chosen based on their distance from the glacier snouts (Figure 1). The distance from each sampled site to the present glacier snouts was 199 m, 297 m, 733 m, 904 m, and 1152 m, respectively. Usually, surface age at each site increases with distance away from the glacier. Ten 50 × 50 cm quadrats were used to sample herbaceous communities at each site. In each quadrat, we recorded coverage, height, and abundance of all plant species. At the same time, we also recorded the height and abundance of bushes and trees on sites number 4 and 5 by using three 4 × 4 m and two 10 × 10 m quadrats. A total of 55 quadrats were examined in this study.

### 3.2. Soil Sampling Analysis

On each site, we collected four samples of surface mineral soil (i.e., top 10 cm). Soil samples were air-dried at ambient temperature and sieved to remove stones and plant debris and then mixed thoroughly to obtain a representative sample. Because the soil texture was uneven, we crushed our sampled soil with a mortar and sieved it. Soil samples (<0.25 mm) were selected and its nutrients were analyzed by chemical analysis methods [35]. Total organic carbon (TOC) was measured by a Flash EA1112 series elemental analyzer (Thermo Fisher Scientific, Waltham, MA, USA). Total nitrogen (TN) and total phosphorus (TP) were digested by the Kjeldahl method [36] and determined by a FLAtstar5000 series flow analyzer (Foss Tecator Ltd., Sweden), while total potassium (TK) was measured by the flame spectrometry standard method [37].

### 3.3. Statistical Analysis

A 64 × 50 (species × quadrats) matrix recording the presence of each species was developed to be the basis of succession analysis. Community type was divided by two-way indicator-species analysis (Twinspan). Principal component analysis (PCA) was used to reveal the relationship among the community groups, and the findings were used to classify the succession stages of the communities. Those analyses were carried out by CANOCO [38] and PCORD [39] computer programs. The differences in top soil nutrients among succession stages were tested by Kruskal-Wallis (*H*) test embedded in SPSS 19.0.

Based on important values (IV) [40] and frequency (F) [41], we classified dominant species and common species. They were calculated using the following equations:

(1) IV=(CR+HR)2,

(2) F=number  of  the  plot  containing  a  speciestotal  number  of  plots×100%,

where, *C*_*R*_ represents relative coverage, *H*_*R*_ stands for relative height. Frequency (F) is the proportion (or percentage) of sample units in which a species occurs.

In one association, species with the highest values of IV and F were assigned to the dominant species category. Excluding the dominant species and those species with the frequency <20% and the lowest IV values, the remainders were classified into the common species category.

In addition, social behavior types (SBT) and the life form of plant species were adopted in this study [42, 43]. Four categories were used in SBT, namely, competitors of natural habitats (C), stress tolerant species (ST), natural pioneers (NP), and weeds (W).

## 4. Results

### 4.1. Classification of Successional Stages

**Figure 2:**
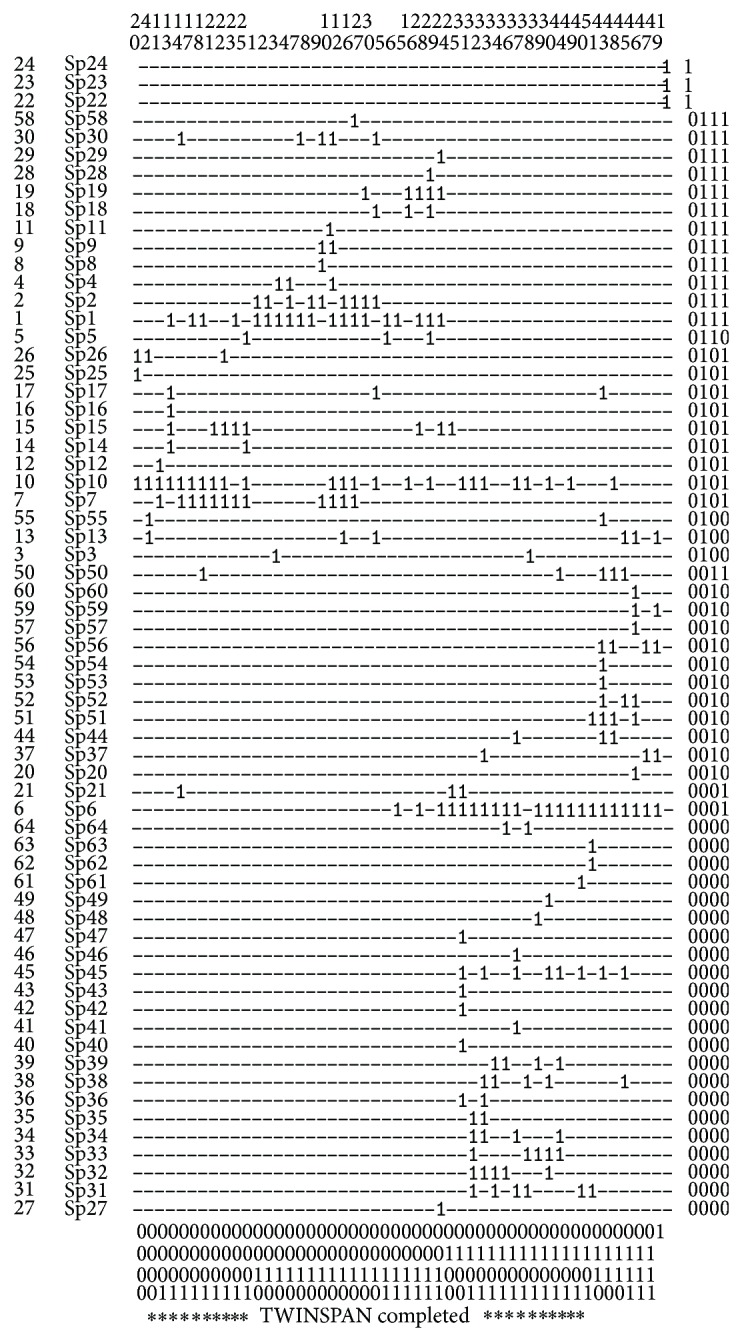
Twinspan analysis of 64 species and 50 plots from the foreland of Glacier-BS1, Mt. Yulong. The Sp + numbers on the left are the species code (Table 5) and the upper numbers are the plot codes. The numbers (0 and 1) on the right and bottom indicate the classification of species and plots.

**Figure 3:**
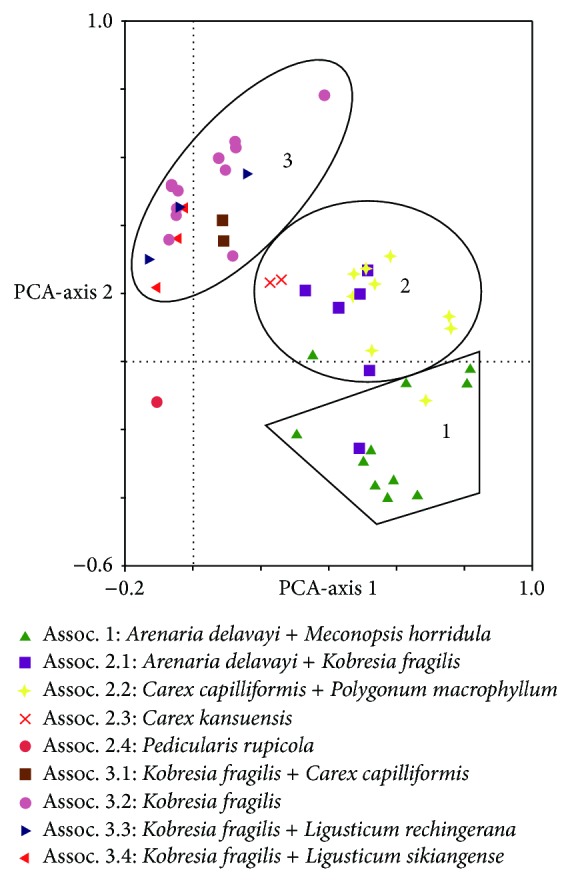
PCA ordination diagram of 50 plots in three succession stages of communities on the foreland of Glacier-BS1 at Mt. Yulong. Assoc. 1–3.4 represent nine associations of herbaceous plants in three succession stages produced by Twinspan.

By using Twinspan Analysis, 50 samples were consolidated into nine groups, representing nine types of herbaceous communities (Table 2; Figure 2). PCA showed that the nine groups could be further grouped into three succession stages (Figure 3). The PCA ordination eigenvalues (*λ*) for the first axis (*λ* = 0.185) was higher than those for the second (*λ* = 0.129) and the third axis (*λ* = 0.109). The first PCA axis represented the succession gradient of time since deglaciation. That is, the succession periods became longer from the right to the left along the first axis in Figure 3. Correspondingly, the community composition, structure, and environment varied greatly from the right to the left, representing the direction of succession. Based on the above data and results, a clear separation of the earliest, middle, and last succession stages could be determined (Figure 3). The average number of species (about 3 per plot) in the earliest succession stage was less than that in the last succession stage (Table 3). The three successional stages were briefly described below.

#### 4.1.1. The Earliest Stage (1)

Twelve plots were put under this stage, which were mainly distributed in S1 and S2 (Figures 1 and 3). Although surface soils were almost absent in those plots, the plants there could still survive on glacial drifts and accumulate soil organic matter. There were fewer common species and smaller plant coverage (2%–10%) in the community. In this stage, the dominant species were* Arenaria delavayi *and* Meconopsis horridula*. Some common species were* Polygonum macrophyllum*,* Carex capilliformis*,* Draba oreodoxa, *and* Cremanthodium smithianum*. All of them are typical vegetation on alpine screes, which are sparsely distributed in rock crevices or on gravelly substrate. Those plants had pubescence or thorns. Given the close proximity to the glacier snouts (about 200–300 m), the growth environment there was unstable and disturbed by ice collapse or rockfall. The presence of* Meconopsis horridula,* which is an annual plant, revealed that the plant community there was still in the initial stage of succession.

#### 4.1.2. The Middle Stage (2)

Similar to the first stage, soil was still almost absent. Glacial tills and weathering products scattered around. A little organic matter was accumulated near plant roots in rock crevices. This stage contained four associations,* Arenaria delavayi + Kobresia fragilis *(6 plots),* Carex capilliformis + Polygonum macrophyllum* (9 plots),* Carex kansuensis* (2 plots), and also* Pedicularis rupicola* (1 plot), which were mainly found in S2 and S3 (Figures 1 and 3). The coverage of the communities was mainly about 10–20%.* Rhododendron phaeochrysum* and* Salix *sp. appeared in shrub layer for the first time. The frequency of* Salix brachista* increased apparently. Yet, they were scattered. Herbaceous plants still dominated this stage. Moreover, some of the dominant species in the middle stage also dominated the earliest or the Last stages, indicating plant succession to be a continuous and gradual process.

*Assoc. Arenaria delavayi + Kobresia fragilis (2.1)*. Two dominant species were* Arenaria delavayi* and* Kobresia fragilis*.* Arenaria delavayi* was also one of the dominant species in the earliest stage.* Delphinium likiangense, Kobresia fragilis*, and* Anemone *sp. were new settlers. Most of the above species were tussock or rosette plants. They adapted to the surrounding environment (i.e., thin soil, poorer nutrient level, and adverse weather conditions) through their special morphological features. 

*Assoc. Carex capilliformis + Polygonum macrophyllum (2.2)*. Dominant species* Polygonum macrophyllum* is also one of the dominant species in alpine meadows in China.* Arenaria delavayi*,* Saussurea spathulifolia, and Salix brachista *were common species in this community. This association contained only one fern of* Sorolepidium ovale*, with its preference to inhabit rock crevices. It is also one of the endemic species on Mt. Yulong. 

*Assoc. Carex kansuensis (2.3).* Species composition was simple in this community, with only five species. The plant coverage of the community varied from 20% to 60%.* Carex kansuensis* was the dominant species, while* Carex capilliformis *was common in this association. These two* Carex *sedges favored the colonization of alpine meadows, showing that this was a transitional community from the earliest stage to the Last stage. 

*Assoc. Pedicularis rupicola (2.4).* This association was composed of three species,* Pedicularis rupicola*,* Aletris pauciflora *var*. khasiana, *and* Arenaria barbata*. The dominant species was* Pedicularis rupicola*, and the vegetation coverage there was about 16%. The difference in species composition might be attributed to the local environment.

#### 4.1.3. The Last Stage (3)

Different from the previous two stages, humus and a litter horizon appeared on the surface over time, showing that soil nutrient had increased significantly. Correspondingly, the vertical structure of the belts was quite complex.* Abies georgei* was the dominant species in the tree layer, and* Larix potaninii *var*. macrocarpa *was occasionally found. The shrub layer was dominated by* Rhododendron phaeochrysum*. There were also some new colonizers such as* Piptanthus tomentosus*,* Rhododendron cephalanthum*,* Rosa omeiensis*,* Berberis dictyophylla*, and* Cotoneaster subadpressus*.* Kobresia fragilis *was the dominant species in the herb layer containing the following four communities,* Kobresia fragilis + Carex capilliformis* (2 plots),* Kobresia fragilis* (12 plots),* Kobresia fragilis + Ligusticum rechingerana* (3 plots), and* Kobresia fragilis + Ligusticum sikiangense* (3 plots). It was distributed in S4 and S5 (Figures 1 and 3). 

*Assoc. Kobresia fragilis + Carex capilliformis (3.1). *The vegetation coverage varied from 15% to 45%, and the dominant species were* Kobresia fragilis *and* Carex capilliformis*. There were five species, namely,* Androsace rigida*,* Pleurospermum hookeri*,* Silene *sp*. 1*,* Meconopsis *sp., and* Saxifraga *sp. They only appeared in this association. 

*Assoc. Kobresia fragilis (3.2).* The total coverage of plant community varied from 40% to 70%. The dominant species was* Kobresia fragilis*, which was a forage plant with enriched nutritional value.* Kobresia fragilis *often became the dominant species in alpine meadows. Compared with the earliest and middle succession stages, the community composition changed greatly in the last succession stage and with many common species in the community, including* Carex capilliformis*,* Saussurea columnaris*,* Youngia *sp.,* Clematis *sp.,* Thalictrum alpinum*,* Phlomis *sp.,* Ainsliaea *sp.,* Potentilla *sp.,* Microula *sp.,* Delphinium *sp*. 3*,* Ligusticum rechingerana*,* Aster vestitus*,* Saussurea fastuosa*,* Ligusticum *sp*., *and* Anemone rupicola*. New colonizers such as* Aster vestitus*,* Anemone rupicola*, and* Plantago depressa *were also found. 

*Assoc. Kobresia fragilis + Ligusticum rechingerana (3.3).* The coverage of the association varied from 45% to 80%.* Kobresia fragilis *and* Ligusticum rechingerana* were the two dominant species, which preferred to colonize alpine meadows. There were common species such as* Saussurea columnaris*,* Saussurea fastuosa*,* Salvia przewalskii*,* Ainsliaea *sp., and* Saussurea vestita*. About 50% of the common species were Asteraceae plants, which were dispersed by wind. 

*Assoc. Kobresia fragilis + Ligusticum sikiangense (3.4).* The coverage of the community changed from 35% to 85%. Again,* Kobresia fragilis *and* Ligusticum sikiangense* were the dominant species. The structure of the association was simple. There were some common species such as* Viola biflora*,* Salvia przewalskii* and* Parasenecio hastiformis*. Meanwhile,* Parasenecio hastiformis *was a new settler and also a hygrophilous plant, revealing that the habitat of this association was wet.

### 4.2. Dispersal Mechanisms of Species

Fruit type, special morphological characteristics, and seed size could explain how seeds were dispersed.  Table 4 shows there were two fruit types of main species (including dominant species and common species) in the deglaciated terrain of Glacier-BS1: dehiscent and indehiscent. Dehiscent fruit was made of a capsule, follicle, and silicle. There were 35.6% of the main species, which had dehiscent fruit. The fruits of the two dominant species in the earliest stage were dehiscent ones (Table 4). Indehiscent fruits include nutlets, achenes, caryopsides, and cremocarps. There were 62.2% of the main species, which had indehiscent fruits. This kind of fruit occupied the biggest component of fruits on the glacial foreland. The fruits of most of the main species in the middle or the last succession stages were indehiscent ones (Table 4). Only a few of the main species had other fruit types.

Also, there were four special morphological features pertinent to dispersal, namely, seeds with wings, pappus, oil, and no appendages (Table 4). Among these, 57.8% of the main species had diaspore without any appendages. The last succession common species had wings or pappus, sharing 33.3% of the main species. Diaspore with oil was the least in proportion. In addition, the size of those seeds was small (<4 mm in length; Table 4). The seeds of* Rhodiola primuloides *were even less than 1 mm in length.

**Figure 4:**
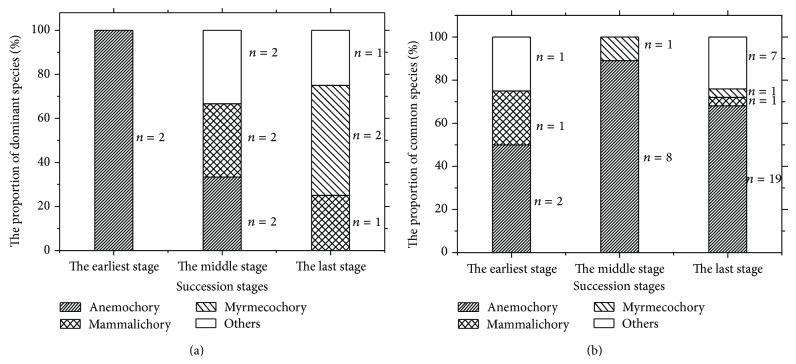
Dispersal modes of dominant species (a) and common species (b) from the earliest stage to the last stage of succession on the foreland of Glacier-BS1.

For species with different fruit types, specific adaptations and seed size [44–46], dispersal methods included anemochory, mammalichory, and myrmecochory on the foreland of Glacier-BS1. Wind-dispersed species in the earliest succession stage contained the dominant species of* Arenaria delavayi *and* Meconopsis horridula* and late successional common species with the same dispersal modes including* Saussurea columnaris, Parasenecio hastiformis*,* Saussurea fastuosa*,* Anaphalis nepalensis*, and* Saussurea vestita*.* Kobresia fragilis *and* Polygonum macrophyllum* were mammalichorous species and dominant in middle and last succession stages.* Ligusticum sikiangense *and* Ligusticum rechingerana* were also dominant in the last stage and were dispersed by ants.  Figure 4(a) indicated that in earlier-stage the seeds of dominant species were dispersed by wind. As community succession progressed over time, more ways of dispersal appeared at specific stages, such as mammalichory and other dispersal methods (e.g., dispersed by water, bird, or human). In the last stage of succession, wind-dispersed species were replaced by zoochorous species (Figure 4(a)). The later one became the dominant species.  Figure 4(b) showed that anemochory was the main form of dispersal among the common species, suggesting that anemochory was the primary dispersal modes throughout the succession process. As succession proceeded, the dispersal methods of the dominant species changed significantly.

## 5. Discussion

### 5.1. Succession Direction and Rate

**Figure 5:**
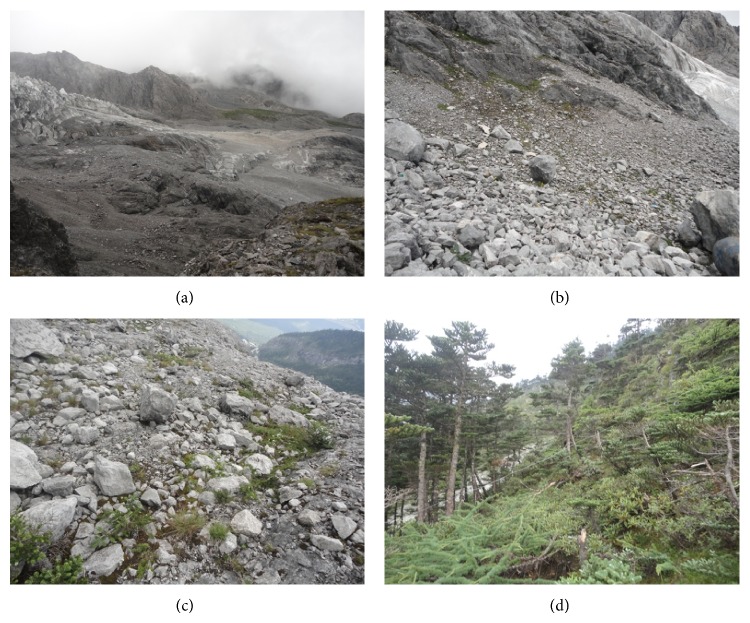
Photos showing the bare land (a), earliest succession stage (b), middle succession stage (c), and last succession stage (d) on the foreland of Glacier-BS1.

Matthews [7] found that numerical approaches were particularly useful when exploring the changes of species composition. Here, the data of plant community succession were analyzed by numerical classification (Twinspan) and ordination techniques (PCA). The direction and trend of plant succession were illustrated clearly in Figures 2 and 3. The plant communities on the foreland of Glacier-BS1 on Mt. Yulong were classified into three succession stages (Figure 3). The earliest successional stage of plant community (at S1 and S2) occurred at an elevation between 4150–4250 m on the glacier foreland (Figure 1). Plant communities (at S2 and S3) in the middle succession stage mainly occurred in the zone between 4000–4150 m a.s.l. (Figure 1). Plant communities (at S4 and S5) of the last succession stage occurred in the zone between 3800–3900 m a.s.l. (Figure 1). Based on the observational data of Glacier-BS1 and related literature, there were three terminal moraines deposited from the glacier in the little ice age between the 17th and 19th centuries. They were located above the elevation of 3800 m a.s.l. [24]. Starting from the late 19th century, the glacier has been retreating rapidly [47]. It retreated about 1250 m between the late 19th century and 1957 [24]. Based on this speed (18.7 m/a), the moraines in the zone between 3900–4000 m a.s.l. might be formed in 1901–1909. Thus, it can be inferred that the earliest, middle, and last succession stages occurred about 9–13, 13–102, and 110–400 years ago, respectively. Specifically, it takes about 110–400 years for the plant communities on the foreland of Glacier-BS1 on Mt. Yulong to proceed from bare land to alpine screes sparse vegetation and then to alpine meadow. Because Figures 5(b)–5(d) is corresponded to the three succession stages in this study, it can be concluded that the last succession stage has developed into fir forest.

Similar study has been conducted on the foreland of Glacier Hailuogou on Mt. Gongga. The succession of plant communities on the forelands of Glacier-BS1 and Glacier Hailuogou proceeds similarly from bare land to fir forests [21]. Plant succession has been reported at higher latitudes (61-62°N) on 39 glacier forelands in the Jotunheim and Jostedalsbreen regions of south-central Norway [17]. In comparison, it was shown that on the glacier forelands below the elevation of 1000 m in the Jostedalsbreen region, the development to birch woodland (Betula-Vaccinium) occurred within 70 years [17]. Although the* Betula* and Ericaceae plants (e.g.,* Betula calcicola *and* Rhododendron phaeochrysum*) on the foreland of Glacier-BS1 also appeared in a similar timespan (13–102 a), they did not dominate in the middle succession stage. The woodland stage on Mt. Yulong occurred about 110–400 years ago because of the higher elevation there (3800–3900 m). Half of the foreland in Glacier-BS1 lies above the local tree line (3800–3900 m), but the glacial foreland in Jostedalsbreen is located below the local tree line (1100–1200 m) [17]. Moreover, on the forelands (1100–1600 m) above the local tree line in the Jotunheim region, the birch woodland stage did not develop after about 250 years. On some sites above 1600 m, the pioneer vegetation (herbaceous plants) still persisted into the mature stage [17], suggesting that the tree line of a region is an important factor determining the change of vegetation types in plant succession on glacial forelands. Furthermore, the tree line also represents a synthesis of environmental factors. That is, the more favorable the environment is, the greater the floristic and structural differences between the early and mature stages are [7], and the more rapid the ecological succession proceeds. Conversely, in the most severe conditions, there was little or nearly no difference in species composition among succession stages [17]. Similar extreme situations existed on a glacier foreland on Ellesmere Island in the high Canadian arctic, in which there was a directional succession pattern without any species replacement [48].

This research showed that natural plant succession takes a very long time. The restoration of primary or secondary bare land can only be speeded up by using appropriate techniques. Particularly for postfire rehabilitation in Mt. Yulong, sowing the seeds of native species might be a desirable method [23], together with other methods such as the seedling of sterile and nonpersistent cereal grains or straw mulch [49], which can prevent soil erosion and improve the ecological restoration ability. The study could serve as a reference for native species selection. The proposed pioneer species are* Meconopsis racemosa*,* Meconopsis horridula*,* Arenaria delavayi*,* Draba oreodoxa*,* Cremanthodium smithianum*,* Polygonum macrophyllum*, and* Carex capilliformis*.

### 5.2. Causes for the Change of Dispersal Mechanism

Plant trait is considered as a comprehensive response of biotic and abiotic environment. Therefore, plant trait (e.g., seed morphological attributes) has been widely examined in ecological research [50–53]. Some scholars have provided evidence about the specific adaptations (e.g., wing or pappus) for dispersal of early colonizers [54–56]. However, the special adaptation may not be a requisite for dispersal by strong winds [7]. Bonde [57] reported that none of the common species on the wind-blown debris of St Mary's Glacier in the Colorado Front Range contain any adaptations to wind. Similarly, the species on the foreland of Glacier-BS1 have no specific adaptations to wind-dispersal in the earliest succession stage. However, because of their small size and special diaspore type (capsule), their seeds are also detached from their fruit by wind [45]. It has been demonstrated that, without any special adaptions, the size and weight of seeds are also strongly correlated with seed dispersal strategy [52, 58, 59]. Because seed mass is believed to be shaped as a size-number compromise [60, 61], according to life form, it could be concluded that early wind-dispersed pioneers (short-lived forbs) often produce lots of smaller seeds, which is in contrast to perennial graminoid relying on zoochory in late successional stage (Table 4). This has been proven by previous studies [62–64]. In addition, Torok et al. [43] further demonstrated the law that the lightest seed weight was selected for natural pioneers (NP) by using quantitative analysis.

Figure 4(a) and Table 4 illustrate that early pioneers are usually dispersed by wind.  Figure 4(b) shows that species with zoochory can colonize the foreland in the earliest succession stage as well. Although this implies that several dispersal methods functioned together in the earliest succession stage, anemochory is obviously the most effective dispersal method (Figures 4(a) and 4(b)). In particular, anemochory is more important on newly-deglaciated terrain, which is far away from well-vegetated areas and also seed sources [65, 66]. With the further increases of terrain age, the dispersal methods of dominant species become more diverse. In the mature stage, zoochory is the main dispersal mode, while wind-dispersal is secondary (Figures 4(a) and 4(b)). Dispersal modes changed in response to the transition of successional stages. This variation can be explained by the principle of competitive exclusion. More specifically, there are only little soil nutrients and a simpler food chain during the nudation period. As plant community appears vertical stratification and becomes more stable during succession, the soil nutrients are enriched slowly (Table 5) and the food chain also becomes more efficient finally. Consequently, there are additional agents for seed dispersal (Figures 4(a) and 4(b)). To survive, plants should make the most use of all available resources, including the diversification of dispersal modes during succession. Then, the most effective dispersal modes are chosen in a new habitat in the last succession stage (Figures 4(a) and 4(b)). Compared with anemochory, zoochory has the advantage of protecting seeds better and dispersing seeds to more suitable habitats. For instance, the feces of herbivores often contain active seeds [67]. After consuming elaiosomes by ants, their seeds can be transported to fertile and moisturized soil, facilitating their germination and seedling establishment [44, 68–70]. For anemochory, it is random with regard to potential safe sites [70], associating with a lower germination rate. Therefore, wind-dispersal often becomes less important in the mature stage (Figures 4(a) and 4(b)).

## 6. Conclusion

1. We analyzed nine types of plant communities on the foreland of Glacier-BS1, which could be grouped into three succession stages. The Earliest succession stage contained only one association, dominated by* Arenaria delavayi *and* Meconopsis horridula*. The middle stage (including Assoc. 2.1, 2.2, 2.3, and 2.4) was dominated by* Arenaria delavayi*,* Kobresia fragilis*,* Carex capilliformis*,* Polygonum macrophyllum*,* Carex kansuensis,* and* Pedicularis rupicola*. The last succession stage (including Assoc. 3.1, 3.2, 3.3, and 3.4) was dominated by* Kobresia fragilis*,* Carex capilliformis*,* Ligusticum rechingerana*, and* Ligusticum sikiangense*. The three succession stages happened about 9–13, 13–102, and 110–400 years ago, respectively. The trend of herbaceous plant succession was from sparse vegetation to alpine meadow. The general tendency was from bare land to herbs and then to fir forests.
2. The main species on the foreland of Glacier-BS1 on Mt. Yulong were dispersed by anemochory, mammalichory, and myrmecochory. Anemochory was the major dispersal mode, especially in the earliest succession stage, in which the dominant species dispersed their seeds by wind. As time went during plant community succession, mammalichory, myrmecochory, and other dispersal modes in dominant species functioned together. Finally, anemochory was replaced by zoochory in the last succession stage.

## Figures and Tables

**Table 1:** Changes of the terminus of Glacier-BS1 since the little ice age (data sources [24, 33]).

Period	Elevation of Glacier End, m	Advance (+) and retreat (−), m
Little ice age (17–19th centuries)	3800	+
—	3900	−214
—	4000	−350
1957	4535	−1250
1982	4100	+800
1997	4200	−150
1998	4150	+
2002	4250	−100
2011	4365	−

**Table 2:** Latin name of herbaceous plants in the foreland of Glacier-BS1 on Mt. Yulong.

Code No.	Latin name of species	Code No.	Latin name of species
Sp1	*Arenaria delavayi *	Sp33	*Clematis *sp.
Sp2	*Meconopsis horridula *	Sp34	*Thalictrum alpinum *
Sp3	*Sedum *sp*. 2 *	Sp35	*Ligusticum *sp.
Sp4	*Cremanthodium smithianum *	Sp36	*Anemone *sp*. 2 *
Sp5	*Pedicularis *sp*. 1 *	Sp37	*Viola biflora *
Sp6	*Kobresia fragilis *	Sp38	*Microula *sp.
Sp7	*Polygonum macrophyllum *	Sp39	*Phlomis *sp.
Sp8	*Caryophyllaceae *sp*. 1 *	Sp40	*Pleurospermum hookeri *var*. thomsonii *
Sp9	*Juncus brachystigma *	Sp41	*Silene *sp*. 1 *
Sp10	*Carex capilliformis *	Sp42	*Meconopsis *sp.
Sp11	*Pedicularis *sp*. 2 *	Sp43	*Saxifraga *sp*. *
Sp12	*Primula dryadifolia *	Sp44	*Saussurea fastuosa *
Sp13	*Ligusticum sikiangense *	Sp45	*Saussurea columnaris *
Sp14	*Sorolepidium ovale *	Sp46	*Caryophyllaceae *sp*. 2 *
Sp15	*Saussurea spathulifolia *	Sp47	*Silene *sp*. 2 *
Sp16	*Draba yunnanensis *	Sp48	*Potentilla *sp.
Sp17	*Delphinium thibeticum *	Sp49	*Euphorbia *sp.
Sp18	*Cyananthus delavayi *	Sp50	*Ligusticum rechingerana *
Sp19	*Rhodiola primuloides *	Sp51	*Saussurea vestita *
Sp20	*Polygonum *sp*. 1 *	Sp52	*Aster *sp.
Sp21	*Anaphalis nepalensis *	Sp53	*Agrostis limprichtii *
Sp22	*Aletris pauciflora *var*. khasiana *	Sp54	*Saussurea centiloba *
Sp23	*Arenaria barbata *	Sp55	*Salvia przewalskii *
Sp24	*Pedicularis rupicola *	Sp56	*Senecio *sp.
Sp25	*Meconopsis racemosa *	Sp57	*Polygonum *sp*. 2 *
Sp26	*Carex kansuensis *	Sp58	*Parasenecio hastiformis *
Sp27	*Androsace rigida *	Sp59	*Fargesia *sp.
Sp28	*Delphinium likiangense *	Sp60	*Plantago depressa *
Sp29	*Anemone *sp*. 1 *	Sp61	*Aster vestitus *
Sp30	*Draba oreodoxa *	Sp62	*Ainsliaea *sp.
Sp31	*Anemone rupicola *	Sp63	*Delphinium *sp*. 2 *
Sp32	*Youngia *sp.	Sp64	*Delphinium *sp*. 3 *

**Table 3:** One-way ANOVA of the average number of species in three succession stages.

Succession stages	The average number of species	*n*	*F*-value	*P*
	Mean ± SD			
The earliest stage	3.75 ± 1.76^a^	12	10.036	0.000235
The middle stage	3.72 ± 1.18^a^	18		
The last stage	6.05 ± 2.10^b^	20		

^*^Data are means ± SD; values with the same letter are not significantly different (*P* > 0.05); values with different letters are significantly different (*P* < 0.05).

**Table 4:** The properties of main species diaspore on the foreland of Glacier-BS1.

Succession stage/category	Fruit type	Specific morphological adaptation	Seed size, mm	SBT	Life form
Pioneers					
*Meconopsis racemosa *	Capsule	No adaptation	1-2	NP	Short-lived forbs
The early stage/dominant species					
*Arenaria delavayi *	Capsule	No adaptation		ST	Perennial forbs
*Meconopsis horridula *	Capsule	No adaptation		NP	Short-lived forbs
Common species					
*Draba oreodoxa *	Silicle	No adaptation	4	ST	Perennial forbs
*Cremanthodium smithianum *	Achene	Pappus	4	ST	Perennial forbs
*Polygonum macrophyllum *	Achene	No adaptation	2.5–3	C	Perennial forbs
*Carex capilliformis *	Nutlet	No adaptation	2	C	Perennial graminoid
The Middle stage/Dominant species					
*Arenaria delavayi *	Capsule	No adaptation		ST	Perennial forbs
*Kobresia fragilis *	Nutlet	No adaptation	2–2.4	C	Perennial graminoid
*Pedicularis rupicola *	Capsule	No adaptation	3	ST	Perennial forbs
*Carex capilliformis *	Nutlet	No adaptation	2	C	Perennial graminoid
*Carex kansuensis *	Nutlet	No adaptation	2	C	Perennial graminoid
*Polygonum macrophyllum *	Achene	No adaptation	2.5–3	C	Perennial forbs
Common species					
*Pedicularis *sp*. 1 *	Capsule	No adaptation		ST	Perennial forbs
*Aletris pauciflora *var*. khasiana *	Capsule	No adaptation	4-5	ST	Perennial forbs
*Cyananthus delavayi *	Capsule	No adaptation	1.8	ST	Perennial forbs
*Arenaria barbata *	Capsule	No adaptation		ST	Perennial forbs
*Meconopsis racemosa *	Capsule	No adaptation	1-2	NP	Short-lived forbs
*Rhodiola primuloides *	Follicle	Wing	0.5–1	ST	Perennial forbs
*Saussurea spathulifolia *	Achene	Pappus	4	ST	Perennial forbs
*Saussurea centiloba *	Achene	Pappus		C	Perennial forbs
*Ligusticum sikiangense *	Cremocarp	Wing, elaiosome		C	Perennial forbs
The late stage/Dominant species					
*Kobresia fragilis *	Nutlet	No adaptation	2–2.4	C	Perennial graminoid
*Carex capilliformis *	Nutlet	No adaptation	2	C	Perennial graminoid
*Ligusticum rechingerana *	Cremocarp	Elaiosome		C	Perennial forbs
*Ligusticum sikiangense *	Cremocarp	Wing, elaiosome		C	Perennial forbs
Common species					
*Saxifraga *sp.	Capsule	No adaptation		ST	Perennial forbs
*Meconopsis *sp.	Capsule	No adaptation		NP	Short-lived forbs
*Viola biflora *	Capsule	No adaptation		ST	Perennial forbs
*Androsace rigida *	Capsule	No adaptation		ST	Perennial forbs
*Delphinium thibeticum *	Follicle	Wing	2–2.5	ST	Perennial forbs
*Silene *sp*. 2 *	Capsule	No adaptation		ST	Perennial forbs
*Anemone *sp*. 2 *	Achene	No adaptation		ST	Perennial forbs
*Anaphalis nepalensis *	Achene	Pappus	1	ST	Perennial forbs
*Saussurea fastuosa *	Achene	Pappus	4–4.5	C	Perennial forbs
*Thalictrum alpinum *	Achene	No adaptation	3	ST	Perennial forbs
*Clematis *sp*. 1 *	Achene	Pappus		C	Perennial forbs
*Ainsliaea *sp.	Achene	Pappus		ST	Perennial forbs
*Saussurea vestita *	Achene	Pappus	4	C	Perennial forbs
*Saussurea columnaris *	Achene	Pappus	3	C	Perennial forbs
*Parasenecio hastiformis *	Achene	Pappus	3-4	C	Perennial forbs
*Saussurea spathulifolia *	Achene	Pappus	4	C	Perennial forbs
*Saussurea centiloba *	Achene	Pappus		C	Perennial forbs
*Aster vestitus *	Achene	Pappus	2.5–3	C	Perennial forbs
*Youngia *sp.	Achene	Pappus		C	Perennial forbs
*Aster *sp.	Achene	Pappus		C	Perennial forbs
*Senecio* sp.	Achene	Pappus		C	Perennial forbs
*Polygonum* sp*. 1 *	Achene	No adaptation		C	Perennial forbs
*Salvia przewalskii *	Nutlet	No adaptation	3	C	Perennial forbs
*Phlomis *sp.	Nutlet	No adaptation		C	Perennial forbs
*Microula *sp.	Nutlet	No adaptation		W	Short-lived forbs
*Agrostis limprichtii *	Caryopsis	No adaptation		C	Perennial graminoid
*Pleurospermum hookeri *var.* thomsonii *	Cremocarp	Wing, elaiosome	3-4	C	Perennial forbs
*Anemone riparia *	Aggregate fruit	No adaptation		C	Perennial forbs

Data source: *Flora of China* (http://frps.eflora.cn/).

**Table 5:** Kruskal-Wallis (*H*) test of soil nutrients during three succession stages in the foreland of Glacier-BS1, Mt. Yulong.

The content of soil nutrition	Earliest stage	Middle stage	Last stage	*F*-value	*P*
The percentage of total phosphorus (TP), %	0.01 ± 0.002^a^	0.02 ± 0.001^b^	0.1 ± 0.014^c^	157.54	0
The percentage of total potassium (TK), %	0.18 ± 0.05^a^	0.23 ± 0.05^a^	0.28 ± 0.09^a^	2.554	0.116
The percentage of total organic carbon (TOC), %	12.13 ± 0.08^a^	12.76 ± 0.13^b^	37.53 ± 2^c^	581.475	0
The percentage of total nitrogen (TN), %	0.02 ± 0.009^a^	0.13 ± 0.035^b^	1.72 ± 0.15^c^	416.819	0

^*^Data are means ± SD; values with the same letter are not significantly different (*P* > 0.05); values with different letters are significantly different (*P* < 0.05).
